# Relapsing faecal incontinence in the elderly: a no man’s land

**DOI:** 10.1186/1471-2482-13-S1-A38

**Published:** 2013-09-16

**Authors:** Gianluca Pellino, Guido Sciaudone, Giuseppe Candilio, Antonio Camerlingo, Rosa Marcellinaro, Federica Rocco, Serena De Fatico, Silvestro Canonico, Francesco Selvaggi

**Affiliations:** 1 Unit of General and Geriatric Surgery, Second University of Naples, Italy

## Background

Post-obstetric neuropathic faecal incontinence is a complex disorder of impaired motor function involving the sphincters and pelvic floor, often associated with impaired rectal evacuation and anorectal anaesthesia [1]. Direct trauma to the anal sphincter complex can give immediate problems or problems later in life. The initial therapy should be conservative. Patients with anatomic sphincter defect or with extensive sphincter damage, muscle loss or pudendal neuropathy are very likely to need surgery. Several surgical approaches have been proposed, but results still remain disappointing, although quality of life seems to be improved [2]; after the initial enthusiasm, the role of artificial bowel sphincter (ABS) has been scaled-down. These problems are even harder to face in elderly patients previously operated on with poor outcome for faecal incontinence.

## Case report

The history of G.A., a 66-year-old woman, affected with faecal incontinence began when she had an obstetric trauma during vaginal delivery, resulting in grade I perineal laceration, managed conservatively. She began suffering with faecal soiling and recurrent diarrhoea. Two years later she had another perineal laceration during vaginal delivery and started complaining for faecal incontinence. After failed conservative therapy, she underwent anterior overlapping external anal sphincter repair, with poor results. She received two ABS implantations and removals because of poor function. The patient experienced intense discouragement and was diagnosed major depressive disorder requiring antidepressants. Implant of a modulator for sacral nerve stimulation (SNS) was advocated but failed. A loop-ileostomy was hence fashioned to allow social functioning.

**Figure 1:**
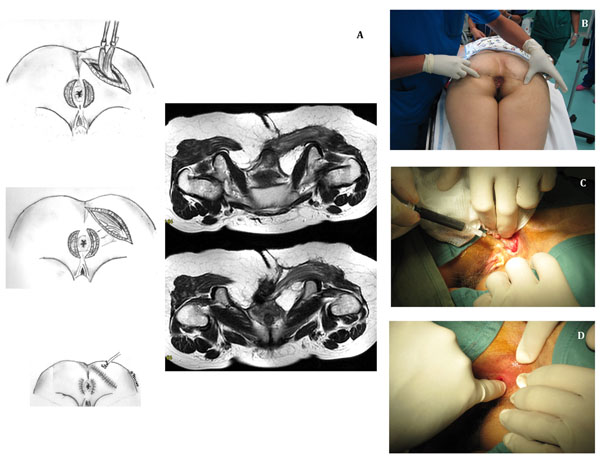
**A.** The drawing represents the intervention: the lower 10% of the right gluteus maximus muscles and fascia are mobilized and distally freed in two strips; these are tunnelled beneath the skin and secured to their contralateral counterparts through lateral incision on the contralateral side of the anus. On the right we compared surgical procedure and an MRI carried on one month later. The fibers of the gluteus maximus can be seen surrounding the rectum therefore providing a neo-sphincter. **B.** Pre-operative picture. The patient is in prone jack-knife position. On the right gluteus a scar from previous surgery is observed. **C.** The Durasphere^®^ is injected in the submucosal space. **D.** Control of bulking effect at the end of the procedure.

When she first came at our observation – at the age of 43 years – she was dejected, but we found her determined in trying to regain trans-anal defecation. We assessed incontinence by means of anorectal manometry, anorectal sensibility, rectal capacity, endorectal ultrasonography and electromyography, concluding for major faecal incontinence with peripheral neuropathy. First, we performed gluteus maximus transposition (GMT). Surgery was performed with the patient in the prone jack-knife position. The muscle on right side was exposed, and superficial fibres were dissected carefully for the remarkable amount of fibrotic tissue from previous interventions to expose the inferior gluteal nerve and vessels, in order to preserve these structures. The free edge of the muscle was split parallel to the muscle fibres, two tunnels were developed from gluteal incision both in front and behind the anal canal to surround the lower rectum and the split gluteus was delivered through these tunnels, suturing the free edges to one another, hence forming a sling around the rectum (Fig. 1). Ileostomy was closed one month later. She achieved good functioning and was asymptomatic for 23 years.

On April 2012 she came at our Unit suffering again from gross faecal incontinence. Function tests revealed a normal ano-rectal physiology. She refused to undergo stoma formation, hence we proposed local injection of bulking agents. We used Durasphere^®^ (*Durasphere*, Advanced UroScience, St Paul, MN, USA), a biocompatible agent composed of pyrolytic carbon-coated beads suspended in a water based carrier gel containing beta glucan, injected in the submucosal plane. The procedure was performed under local anaesthesia. Three applications were carried out, at intervals of 3 months. Patient is actually been followed-up twice a month, and she has gained good continence, allowing her a good social functioning and quality of life; at 12-month follow-up results seem stable with time.

## Discussion

In adult women, faecal incontinence is most commonly caused by obstetric trauma. Thirty-five percent of primagravida vaginal deliveries can result in sphincter injuries, but some women may remain asymptomatic [3]. Moreover, there is a physiological decrease in sphincter function in older individuals, which may lead to worse bowel control [3].

Surgical treatment of post-obstetric faecal incontinence usually consists of sphincter repair if there is clinical or ultrasonography evidence of sphincter disruption or failed SNS, reserving post-anal repair for the treatment of incontinence in patients with history of traumatic or prolonged labor associated with physiologic evidence of pudendal nerve injury, low anal canal pressures, anal anesthesia, and prolonged pudendal nerve conduction time.

Striated muscle transposition has included the gracilis muscle and the gluteus maximus [4]. The gracilis sphincterplasty became more popular because of the accessibility of this muscle. Unfortunately, the results of gracilis muscle transposition without electrical stimulation are poor: because sustained contraction is not possible and long-term electrical stimulation is needed to convert the rapid twitch-fatigue striated muscle to a slow-twitch sphincter, the combined approach with electrical stimulation has gained popularity for the treatment of faecal incontinence; however, results have been disappointing [4]. GMT is not widely used for treatment of post-obstetric faecal incontinence, but physiologic studies suggest that the procedure may improve resting and squeeze anal canal pressures and elongate the high pressure zone [4,5]. We opted for GMT as the neurovascular pedicle is reported to undergo less traction after transposition compared with the graciloplasty based on cadaver studies, moreover gluteus muscle transfer far exceeded the amount of muscle tissue of a normal anal sphincter despite muscle atrophy after transposition [5]. Moreover, GMT is reported to induce a double curvation of the anal canal in contrast to the graciloplasty, enhancing the natural ano-rectal angle [5].

Management of patients after surgical failures still remains a no-man’s land.

After excluding functional disturbances, we advocated injection of bulking agent: this procedure is reported to be well tolerated, and results seem stable with time. The incidence of adverse effects (mainly bleeding and infection of application site) is negligible [6].

Dealing with elderly patients with faecal incontinence who failed previous surgical approaches can really be challenging and frustrating for both patient and surgeon. Patient selection is the key. The patient should really be motivated in avoiding a faecal diversion; on the other hand, a treatment as conservative as possible should be provided. Injection of bulking agents is safe and effective. Our patient gained a much better quality of life, as good continence was achieved.

It is necessary to cautiously approach such individuals, considering critically whether our intervention could give advantages and better quality of life.

Further investigations on the topic are demandable.

## Competing interests

Authors have no competing interests to disclose.

## Authors’ contributions

GP designed the study, and wrote the draft of the manuscript. GS, SDF and GC collected data and participated in the drafting. SDF, AC, RM and FR collected and analyzed data. SC and FS conceived of the study, participated in its design and coordination and helped to draft the manuscript. GP made the drawing in figure 1. FS performed surgical procedure in our Unit as operating surgeon. All authors read and approved the final manuscript.
